# Does personality matter: examining the value of personality insights for personalized nudges that encourage the selection of learning resources

**DOI:** 10.3389/frai.2024.1211142

**Published:** 2024-07-16

**Authors:** Pedro Cardenas Canto, Vania Dimitrova, Stuart Sherman, Stuart W. Flint

**Affiliations:** ^1^Scaled Insights, Nexus, University of Leeds, Leeds, United Kingdom; ^2^School of Computing, University of Leeds, Leeds, United Kingdom; ^3^School of Psychology, University of Leeds, Leeds, United Kingdom

**Keywords:** personalization, personality insights, nudging, resource recommendation, learning

## Abstract

Nudging is a mechanism aimed at influencing people's behavior while maintaining the individual's freedom of choice. Nudges have been adopted in learning contexts where individuals are responsible for shaping their learning and, at the same time, receive guidance from the system. Not everyone responds to nudges in the same way. While social science research indicates that individual differences play a crucial role in peoples' nudgeability, there has been little research examining computational approaches that explore how individual differences affect user responses to nudges (especially in a learning context). Two studies were conducted to explore how individual differences, specifically focusing on personality, can affect nudge response in the context of healthcare education, where individuals use resources as a part of their informal learning and professional development. Different nudges, designed based on personality characteristics, were provided to draw individual users' attention to educational resources to encourage user engagement. The findings indicate that personality insights can be a predictor for nudge selection, suggesting that different nudges may be more effective when recommending learning resources to people with different personality characteristics.

## 1 Introduction

Humans are exposed to and make decisions about a vast amount of information which is processed consciously and subconsciously. The amount of data humans are exposed to, and subsequently process daily, has grown exponentially with the proliferation of digital devices both in-home and at work, such as via desktop computers, laptops, and mobile devices. The wide variety of data that flows on electronic channels, along with imprecise and ambiguous information, may lead to confusion or frustration. During the coronavirus (COVID-19) pandemic, online learning platforms were shown to be efficient in supporting training and education globally,^1^ where the demand for digital services increased significantly.^2^ The subsequent abundance of educational resources makes it challenging for individuals to decide what resources to follow. This can have a negative impact, especially when resources are used for informal learning and professional development.

One method to support users to navigate educational resources is to provide pointers, or nudges, to relevant content. However, educating individuals through online resources is a challenging task because a combination of processes that are involved, such as adequate strategic preparation, thinking in processes, and the amalgamation and reinforcement of all parties involved (Adedoyin and Soykan, 2020). While there is an abundance of recommender systems, where the user is directed to relevant content, there is less work on recommending resources for educational purposes to help individuals engage with content. Crucially, recommendations can be challenging when resources are used for informal learning and learners can have different goals and motivations. In such cases, it is important to foster self-regulation and preserve the learner's “freedom” to explore the information space and decision to read the resource.

To address these challenges, we propose the use of nudges to increase uptake of resources among learners. Nudges refer to subtle changes to choice architecture that can influence behavior without altering the autonomy of choice (Thaler and Benartzi, 2004; Thaler and Sunstein, 2008). These subtle changes, or cues, can be added to resource recommendations to 'nudge' the user to that resource while maintaining the user's freedom of choice, which is crucial for self-regulation and independent learning (Weijers et al., 2020). Nudging has been adopted in various scenarios to improve the decision-making process. For instance, default choices have shown to be effective in increasing organ-donor consent (Weijers et al., 2020) and incentives promoting smoking cessation (Giné et al., 2010). Another example is to use nudges to encourage the selection of healthier food options in school cafeterias and eating habits among children (Hanks et al., 2012). Nudging in educational settings and domains is a relatively unexplored area, which is gaining popularity with the rapid growth of digital education (Damgaard and Nielsen, 2018; Dimitrova and Mitrovic, 2021).

Designing nudges and creating an effective choice architecture for reading educational resources is a complex task due to the wide-ranging factors that influence people's responses to behavioral interventions. Pre-existing preferences (De Ridder et al., 2022) or personality insights (Warberg et al., 2019) are some of the avenues that can be considered to create personalized or tailored nudges. However, to date, no research has examined whether and how these characteristics can be used to design nudges as part of digital learning environments.

The current study explored how individual differences, specifically focusing on personality, can affect the use of nudges for learning. The context of the study is provided by Health Education England^3^ (HEE), a national organization which provides education and training for the healthcare workforce in England through a wide range of e-learning programs.^4^ HEE faces the challenge of motivating and supporting a vast and diverse group of health professionals in engaging in self-studies and completing their e-learning activities alongside their regular workload. This is especially challenging in highly demanding and stressful jobs in the healthcare sector. It is expected that a personalized resource recommendation approach could facilitate the selection of the educational resources that HEE provide. At the same time, preserving the explorative nature of the educational resource space and offering minimal guidance to encourage user engagement is of paramount importance. Consequently, we explored the use of nudges to extend the existing HEE learning resource recommendation.

We present two studies that address the following research question: “*Can personality insights be used as a means of informing the use of nudges for recommending educational resources?*” We investigated the effectiveness of five types of nudges in an e-learning environment (HEE's e-learning for health platform), which recommends resources for self-learning and professional development in healthcare. Specifically, the study examined the effect of personality insights on selecting nudges. The importance of studying personality insights is in line with other investigations that unveil their crucial role in learning and predicting success during educational careers (Raad and Schouwenburg, 1996; Nießen et al., 2020). To complement the robustness of this analysis, the Behavioral Artificial Intelligence (AI) approach developed by Scaled Insights that measure personality insights and have been successfully tested in other contexts (Flint et al., 2020, 2021) is used to explore its applicability in the educational resource domain. In a broader context, the study presented here contributes to a new avenue for creating choice architectures and nudges that could be used to encourage e-learning and offers a case study in the healthcare domain.

## 2 Related work

### 2.1 Use of nudges in learning

A major obstacle to the success of e-learning, especially for informal learning, is learners' motivation and engagement (Dhawan, 2020). Providing interventions that can motivate individuals and promote engagement could facilitate the learning process (Damgaard and Nielsen, 2018). Previous research has examined the impact of different nudge interventions on the education context. Table 1 describes five behavior change interventions that have been employed within education.

**Table 1:** Nudge Interventions implemented in education.

**Intervention**	**Description**	**References**
Default	The choice explored the impact of opt-in and opt-out techniques. Studies have tested the default intervention in the education sector. Marx and Turner (2017) reported that students who received offerings to loans are more likely to borrow the default amount than those who did not receive such an offer. They also highlighted the positive effects on earned credits and the grade point average. Bergman and Rogers (2017) examined the adoption of opt-out vs. opt-in default regarding text messaging to keep parents informed about their children's performance at the high school level. Their findings revealed that parents who opted in were more engaged with the school and aligned with high-performing students.	(Bergman and Rogers, 2017; Marx and Turner, 2017)
Framing	The choice involved small, deliberate changes to the choice environment. Fryer et al. (2012) analyzed the effect of framing teacher performance incentives as a loss. They were paid in advance and asked to return the payment if their pupils did not improve their results. Study outcomes suggested that if incentives were provided over a longer time period, teachers responded better by restructuring their strategy together with their efforts. Wagner (2017) explored the loss and gain frames in education. Children were given either a zero endowment of test points with the opportunity to get more points by answering correctly or skipping questions. By contrast, children were offered a second different scheme, a positive endowment that was deducted due to wrong or omitted answers. The results suggested that children with higher abilities were likely to earn points on the loss scheme than those with low abilities, which made fewer points.	(Fryer et al., 2012; Wagner, 2017)
Deadlines	The intervention implemented deadlines as a means of avoiding procrastination. This type of intervention has been extensively analyzed in the education field. Studies (O'Donoghue and Rabin, 1999a,b; Ariely and Wertenbroch, 2002) examined the effect of deadlines as a commitment device for students to study and complete their activities sooner rather than later.	(O'Donoghue and Rabin, Incentives for procrastinators., 1999a; O'Donoghue and Rabin, 1999b, Doing it now or later; Ariely and Wertenbroch, 2002)
Rewards (Extrinsic Motivation)	The application of extrinsic motivation in the education field has been studied extensively. For instance, Guryan et al. (2016) investigated the effect of non-monetary rewards on students who were offered incentives to read books over the summer holiday. By completing such an activity, pupils could earn points which could be spent on different items such as board games and sports equipment. Karlsen and Varhaug (2016) proposed a different approach and researched the effects of incentives targeted at university students. They examined whether students who were offered to enter a lottery to win books to support their studies were more likely to complete the enrolment process. The results suggested that such an incentive was ineffective since the aforementioned group of students was not more likely to complete the enrolment procedure than those who decided to ignore entering the lottery.	(Guryan et al., 2016; Karlsen and Varhaug, 2016)
Goal setting	The intervention investigated setting goals as a means of commitment and improving educational outcomes. Clark et al. (2017) examined the effect of university students' self-set, task, and performance goals. The outcomes indicated that task-based goals led students to commit and achieve better exam results. Lent and Souverijn (2017) also investigated the impact on grades focused on a performance-based goal strategy in university students. According to their results, such an intervention positively affected students who initially performed poorly.	(Clark et al., 2017; Lent and Souverijn, 2017)
Visualization and prompts	The intervention explored the potential for video-based learning to provide engaging and effective learning environments in education. The nudges used include signposting, in the form of interactive visualizations, and personalized prompts to encourage engagement with videos. Dimitrova and Mitrovic (2021) present several user studies that examined the effectiveness of personalized nudges tailored to an individual's interest in specific aspects of the video rather than individual differences. They compared two versions of the systems with and without nudges and showed that the nudges had positive impact on noticing key parts in the video and writing comments.	(Dimitrova and Mitrovic, 2021)

None of the studies presented in Table 1 considers personality a core element when designing the nudges for learning. The effectiveness of nudges for decision-making depends on cognitive factors and personality differences (De Ridder et al., 2022). Studying individual differences based on personality insights can inform how to tailor nudges for improved engagement with learning content and could highlight potential insights about applying nudges to specific user sub-clusters (Ingendahl et al., 2021). This study investigates the effect of personality insights on responding to nudges when educational resources are recommended.

### 2.2 Persuasion

Adding nudges for engaging with educational resources can be linked to persuasion. According to Fogg (2003), the purpose of persuasion is to modify attitudes and behaviors through technological interaction without coercion or deception. Furthermore, persuasion alludes to the communication procedure where there is an interaction between the persuader and the persuade aimed at changing the recipient's attitude or behavior (Harjumaa and Oinas-Kukkonen, 2007). Harjumaa and Oinas-Kukkonen (2007) suggest that the interaction process has three types of persuasion: interpersonal, computer-mediated, and human-computer persuasion.

Interpersonal persuasion takes place when two or more individuals interact with each other. Computer mediation occurs when people are persuaded by others through computer technologies such as email or instant messages. Finally, human–computer persuasion refers to how people are persuaded when interacting with computer technology. The value of this last type of interaction is that social communication is possible (Nass et al., 1994), which is how humans communicate, and its link with technology refers to building trust between people and interfaces, also called persuasive technology. Furthermore, persuasive technology refers to technology that has been designed to persuade individuals to change their attitudes or behaviors in a desirable direction (Fogg, 2003).

Recent study in persuasion explores the relationship between personality and persuasive features in mental health applications (Alqahtani et al., 2022). It showed that perceived usefulness of various persuasive features can be linked to personality traits and may also have some domain dependency. Similarly, Ndulue et al. (2022) showed that personality links to the perceived persuasiveness of different behavior change strategies used in gamified applications. Additionally, Fatahi et al. (2023) reported that personality affects the receiving of persuasive messages in music recommendation, which suggests that personality can play a role when tailoring persuasive messages in a music domain. In the tourism domain, Alves et al. (2023) showed that personality can be used to predict the choice of tourist attractions and travel-related preferences, and concerns and some personality traits can also predict motivation.

In the current study, the human–computer persuasion was analyzed in the light of the interaction between individuals (healthcare professionals, medical students including physicians, nurses, midwives, social workers, and radiographers) and the likeliness of selecting a resource. The persuasion features were in the form of nudges. The link between perceived effect of the nudges (willingness to read a learning resource) and personality was examined in the context of healthcare education.

### 2.3 Personality insights

Personality insights^5^ refer to the combination of behavior, emotion, motivation, and thought patterns that define an individual and have been shown to influence human behavior (Ferwerda et al., 2019; Logan et al., 2019). Different models have been created to categorize personalities, such as measures of the well-known Big Five (Donnellan et al., 2006) and Basic Human Values (Bilsky and Schwartz, 1994; Fetvadjiev and He, 2019). The Big Five model (McCrae and John, 1992) measures personality through five dipolar scales, namely, Extraversion, Neuroticism, Agreeableness, Conscientiousness, and Openness (see Table 2 for a detailed description). The Five-Factor model has been used in different contexts, for example, to examine the impact of consumer personality on preferences toward particular brands (Mulyanegara et al., 2009).

**Table 2:** Personality models used in different contexts adapted from the study by Mulyanegara et al. (2009) and Schwartz (2012).

**Insight**	**Description**	**Model**
Extraversion	Person's tendency to seek stimulation in the company of others	Big Five
Neuroticism	The extent to which a person's emotions are sensitive to the person's environment	
Openness	The extent to which a person is open to experiencing different activities	
Conscientiousness	Person's tendency to act in an organized or thoughtful way	
Agreeableness	Person's tendency to be compassionate and cooperative toward others	
Openness to change	Emphasizes independent action thought, feeling, and readiness for new experiences	Basic Human Values
Self-enhancement	Seeks personal success for themselves	
Conservation	Emphasizes self-restriction, order, and resistance to change	
Self-transcendence	Shows concern for the welfare and interests of others	
Hedonism	Seeks pleasure and sensuous gratification for themselves	

The theory of Basic Human Values refers to the characterization of cultural groups, societies, and individuals to trace change over time and explain the motivational bases of attitudes and behavior (Bilsky and Schwartz, 1994; Schwartz, 2012) through the study of five items, namely, Openness to Change, Self-Enhancement, Conservation, Self-Transcendence, and Hedonism (see Table 2). The evaluation of the relationship between values and voting behavior (Barnea and Schwartz, 2008; Tatarko, 2017) is an example of how values could be used during electoral choices.

The integration of personality insights and **nudges** as a critical partnership for decision-making has been studied in several cases. For instance, the analysis of job performance through the lens of personality inventories such as the Big Five has demonstrated its effectiveness in influencing behavior (Barrick and Mount, 1991). In the security context, Acquisti et al. (2018) have analyzed the impact of personality interventions and tailored nudges to influence participants' disclosure choices. The case of education receives special treatment since, according to Szaszi et al. (2018), only 4% of the studies in nudging were related to such a field. By contrast, 42% of the analyses were associated with promoting health. This highlights the importance of studying the application of personality insights in nudging. Although the effect of personality on learning contexts has been studied (e.g., Kobayashi et al., 2023 show that personality can play a role in the effectiveness of group learning), the value that personality insights can add to nudges for recommending resources has not been explored previously.

### 2.4 Persuasive design, nudging, and personality insights

Linking persuasive design, nudges, and personality insights can help outline their contribution to the nudge selection process. In the first instance, the relationship between persuasive design and nudging is grounded in the change-oriented feature because both factors are aligned to achieve the same essential objective of influencing behavior change. Nonetheless, Segerståhl and Oinas-Kukkonen (2007) described that persuasive design is associated with attitudes and behavior change, whereas nudging is linked to decision-making (Mirsch et al., 2017). More recently, El Majjodi et al. (2022) showed that adding additional information when recommending products, in this case front-of-package nutrition labels to recommend recipes, can reduce the choice difficulty which can affect positively the user experience with the system.

**Figure 1:**
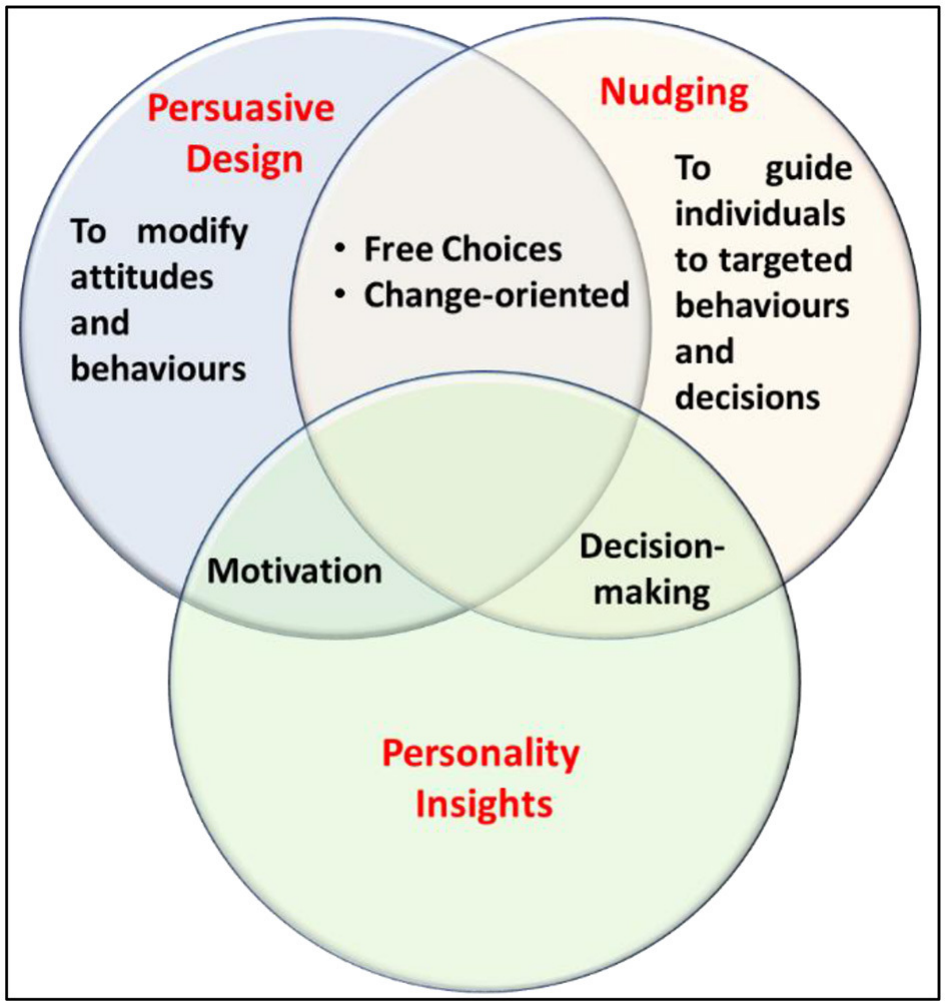
Relationship between persuasive design, nudging, and personality insights, which follows the classification in the study by Castmo and Persson (2018) and adds personality insights.

Figure 1 illustrates the relationship between personality insights described previously under two main aspects—first, to modify attitudes and behaviors, and second, to guide individuals toward targeted behaviors and decisions. The association between personality and the other pair of characteristics, persuasive design and nudging, unveils two core aspects. As depicted in the study by Marchiori et al. (2017), the nudging theory has roots in psychology and helps explain behavioral decision-making. Persuasion is closely related to individual differences, which leads to understanding human motivation (Deci and Ryan, 1985; Ryan and Deci, 2000) (see Figure 1).

The importance of personality insights in addressing problems linked to changing the behavioral component has been tested in different cases. Several models, such as the Big Five or Basic Human Values (see Section 2.3), have been used to improve or predict people's decisions. However, to the best of our knowledge, the use of multiple personality insights to investigate nudging for e-learning has not been explored previously. The current study was intended to fill this gap by exploring 113 personality insights aimed at creating a rich personality profile of the users and then examining their predictive effects on nudges for engaging with educational resources in healthcare education.

## 3 Methods

### 3.1 Selected nudges

Five nudges were selected from the choice architecture categories and techniques based on the review by Münscher et al. (2016) and taxonomy of choice architecture. The selected nudges (see Table 3) correspond to common ways of recommending resources for learning.

**Table 3:** Choice architecture categories and techniques implemented in the nudges offered in this study, which is adapted from the study by Münscher et al. (2016).

**Category**	**Technique**	**Justification**
Decision information	Feedback	Make own behavior visible. Feedback can have a powerful influence on behavior and contribute to self-optimization.
	External information	Make information visible. Making visible external decision-relevant information can empower decision-makers. For this study, the core information is about external entities in the healthcare sector.
	Opinion leader	Provide a social reference point. The behavior of other individuals can appear in the form of group behavior, which is appreciated for particular motivations, such as knowledge, fame, or a specific function. In this investigation, the information about well-known organizations in the healthcare domain will be added to work as role models.
Decision structure	Benefit	Connect decision to benefit. Linking a desired behavior to a small benefit can change the probability of occurrence and may trigger additional costs or benefits. The present investigation will show the potential benefits of selecting this technique to final users, such as earning points (kudos).
Decision assistance	Self-commitment	Facilitate commitment. Commitment toward certain behaviors makes people more likely to follow through since it compensates for self-control issues. By selecting this technique, users will self-impose deadlines for completing the activity.

The choice of the set of nudges (Feedback, External Information, Opinion Leader, Benefit, and Self-commitment) is grounded in established principles of autonomy-preserving nudging and substantiated by different research findings. Van Roekel (2023) emphasized the nuanced relationship between nudges and autonomy, showcasing how nudges can be thoughtfully designed to respect individuals' autonomy while remaining potent in influencing behavior. This nuanced approach is acknowledged and embraced in the present study integrating several nudges. For instance, Feedback, as highlighted by Van Roekel (2023), makes learners' behavior visible, promoting self-optimization-a principle that inherently respects autonomy. Similarly, External Information's provision of visible, relevant external information is instrumental in empowering decision-makers to make informed choices, a practice that harmonizes with the principles of autonomy preservation, especially within an educational context where informed choices are vital. Van Roekel (2023) also noted the potential of Opinion Leader nudges, where the presence of a role model positively influenced academic achievement. This resonates with our selection, as our Opinion Leader nudges utilize the behavior of well-known healthcare organizations as role models to motivate learners, acknowledging the positive impact that such influencers can have on behavior without undermining autonomy. In addition to the insights from Van Roekel (2023), Zamprogno et al. (2020) examined the use of nudges in computer science courses to improve student learning strategies, which supports our approach to utilizing Feedback and Self-commitment nudges. Zamprogno et al. (2020) demonstrated that carefully designed nudges about where students should focus their efforts can enhance how students act on generated feedback, effectively preserving their autonomy while aligning their actions with the learning goals intended by the course staff. By considering these findings alongside the autonomy-preserving framework of Van Roekel (2023), our study aimed to contribute to the evolving understanding of how autonomy-respecting nudging can positively impact students' engagement with educational resources.

### 3.2 Detection of personality insights

This work used the Scaled Insights' proprietary Behavioral Artificial Intelligence (AI) tool^6^ to analyze personality insights, based on which we can identify the effect of personality insights on the selected nudges for learning. The robustness and effectiveness of this technological tool to extract personality insights have been examined in several studies as a means of clustering personality attributes. The results have been used to predict behaviors and outcomes including awareness, attitudes and actions of people identified as at high risk of severe illness from COVID-19 infection (Flint et al., 2020), as well as the experiences of people living with obesity accessing healthcare in England (Flint et al., 2021).

**Figure 2:**
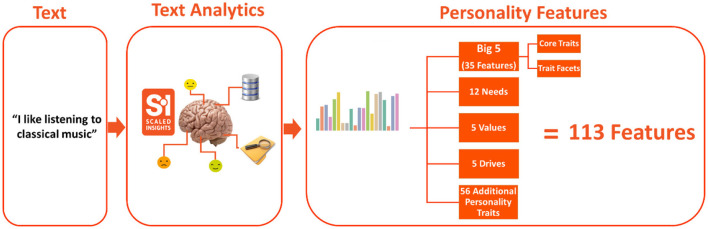
Scaled Insights' proprietary tool data flow architecture.

The Scaled Insights' Behavioral AI tool functions by meticulously analyzing textual data, encompassing written content, with the goal of deriving intricate personality insights from the text. Employing sophisticated natural language processing (NLP) techniques, it delves into linguistic cues, dissects writing style nuances, and scrutinizes content details, thus extracting a comprehensive set of features corresponding to a remarkable 113 personality features, combining different models such as Big Five and Human Values (Roccas et al., 2002). This in-depth analysis encompasses the examination of word choices, the structure of sentences, and the nuanced emotional tones embedded within the text, culminating in the creation of an individualized personality profile. This profile provides invaluable information about a broad spectrum of traits, including but not limited to openness, extraversion, conscientiousness, drives, needs, values, thinking styles, and sentiments. These insights, meticulously gleaned through the Scaled Insights' Behavioral AI, enable professionals to facilitate and support patients in adhering to health-promoting behaviors or making well-informed decisions, leveraging the power of effective nudges and personalized communication. Furthermore, the Scaled Insights' Behavioral AI tool extends its utility beyond mere profiling, and it constructs predictive models that forecast how an individual's personality is likely to influence their behaviors and eventual outcomes, providing a holistic understanding that empowers professionals to optimize their interventions and support strategies (see Figure 2, Table 4).

**Table 4:** Personality profile characteristics of the insights derived from scaled insights' behavioral AI.

**Feature**	**Description**
Emotionality	Aware of your feelings and how to express them.
Modesty	Uncomfortable being the center of attention.
Social Group Orientated	Measures the degree to which a person's values and behaviors are rooted in their sense of family.
Distractible	Feel your desires strongly and are easily tempted by them.
Self-discipline	Can tackle and stick with tough tasks.
Openness	The extent to which a person is open to experiencing different activities.
Anxiety	Tend to worry about things that might happen.
Vulnerability	Easily overwhelmed in stressful situations.
Workhorse	Measures the degree to which a person has a strong work ethic vs. preference for leisure and non-work activity.
Grounded	Exhibits groundedness and a desire to hold things together. They need things to be well organized and under control.
Happiness	Measures the degree to which a person is optimistic, upbeat, and happy.
Neuroticism	Measures the degree to which a person expresses strong negative emotions.
Aggressive	Measures the degree to which a person exhibits anger or aggression.
Cold	Measures the degree to which a person is emotionally unresponsive and has difficulty empathizing with others.
Depression	Measures the degree to which a person may have difficulty finding joy in their life.
Body focus	Measures the degree to which a person focuses attention on their body or other people's bodies.
Persuasive	Measures the degree to which a person can create rapport to persuade others.
Extraversion	A person's tendency to seek stimulation in the company of others.
Neuroticism	The extent to which a person's emotions are sensitive to the person's environment.
Openness	The extent to which a person is open to experiencing different activities.
Conscientiousness	A person's tendency to act in an organized or thoughtful way.
Agreeableness	A person's tendency to be compassionate and cooperative toward others.

### 3.3 Experimental design

#### 3.3.1 Aim and research question

To investigate the effect of personality insights on designing nudges for recommending educational resources, we conducted two **user studies** that followed the same experimental setup. The research presented here is a continuation of a previous study that focused on evaluating the perceived effectiveness of different choice architecture techniques (nudges) in an e-learning environment for people dedicated to the healthcare context (Piotrkowicz et al., 2020). The previous analysis was aligned with the challenges faced by HEE. As part of the first user study, the effectiveness of five different nudges (see Table 3) in medical students at the University of Leeds was examined by considering demographic variables. The results were not conclusive due to the small sample size. A second user study was conducted with healthcare professionals following the same experimental design to investigate further the effect of the same nudges (Table 3), to encourage learners to use the e-learning resources offered by HEE.

Throughout the study, we refer to the research presented here as one study which de facto combines data from the two user studies:

- User study 1—medical students from the University of Leeds
- User study 2—healthcare professionals at the Leeds Teaching Hospitals NHS Trust

Combining data from both user studies, we address the following research question:

*Can personality insights be used to design nudges for recommending educational resources?*

Ethical approval was obtained from the Faculty of Medicine and Health, University of Leeds, UK for both studies. For Study 2, which involved health professionals, ethical approval was obtained also from the UK NHS Research Ethics Committee.

#### 3.3.2 Procedure

Both user studies were implemented as an online survey and followed the same experimental setup, which included several parts.

**Part 1**. Information sheet and consent form. The participants were presented with the experimental setup and asked for consent to participate in the study.

**Part 2**. Demographic information. The participants were asked questions about their occupation, age, years of experience in healthcare, and use of technology for learning.

**Part 3**. Open-ended questions. These questions were aimed to collect free-text samples that enclose personal opinions about specific topics. The questions were adapted to the specific user groups and were created with the help of health educators.

User study 1, medical students, included the following open-ended questions:

- Why did you choose your degree? (You may want to write about: motivations, aspirations, role models, dreams, rewards, peers, family, society, lifestyle, benefits, and many others)
- What do you like most about Leeds? (You may want to write about: location, people, food, entertainment, nature, culture, transport, architecture, history, opportunities, business, and many others)
- Describe a challenge you have faced that relates to your profession. Why was it difficult for you and how did you overcome it? (You may want to write about the problem, solution, people involved, your reaction, your feelings, and many others. Please do not disclose any sensitive or identifiable information.)
- How do you think technology will change your professional role over the next 10 years? (You may want to write about patients, healthcare professionals, organizations, different contexts and locations, conditions, impacts, and many others)
- If you had an unlimited budget to revolutionize healthcare, what would you do? (You may want to write about patients, healthcare professionals, organizations, different contexts and locations, conditions, impacts, and many others).

User study 2, healthcare professionals, included the following open-ended questions:

- Why did you choose your profession? (You may want to write about: motivations, aspirations, role models, dreams, rewards, peers, family, society, lifestyle, benefits, and many others)
- What do you think the long-term impact of the pandemic will be on the way that you access education and training? (You may want to write about: ways of accessing training, the topics that you choose, use of technology, and many others)
- Describe a challenge you have faced that relates to your profession. Why was it difficult for you and how did you overcome it? (You may want to write about the problem, solution, people involved, your reaction, your feelings, and many others. Please do not disclose any sensitive or identifiable information.)
- How do you think technology will change your professional role over the next 10 years? (You may want to write about patients, healthcare professionals, organizations, different contexts and locations, conditions, impacts, and many others)
- If you had an unlimited budget to revolutionize healthcare, what would you do? (You may want to write about patients, healthcare professionals, organizations, different contexts and locations, conditions, impacts, and many others).

The answers to the open-ended questions provided free text with opinions (minimum size of 500 words all together for the open-ended questions was imposed). The text was used as an input to the Scaled Insights' Behavioral AI tool (see Section 4.2).

**Figure 3:**
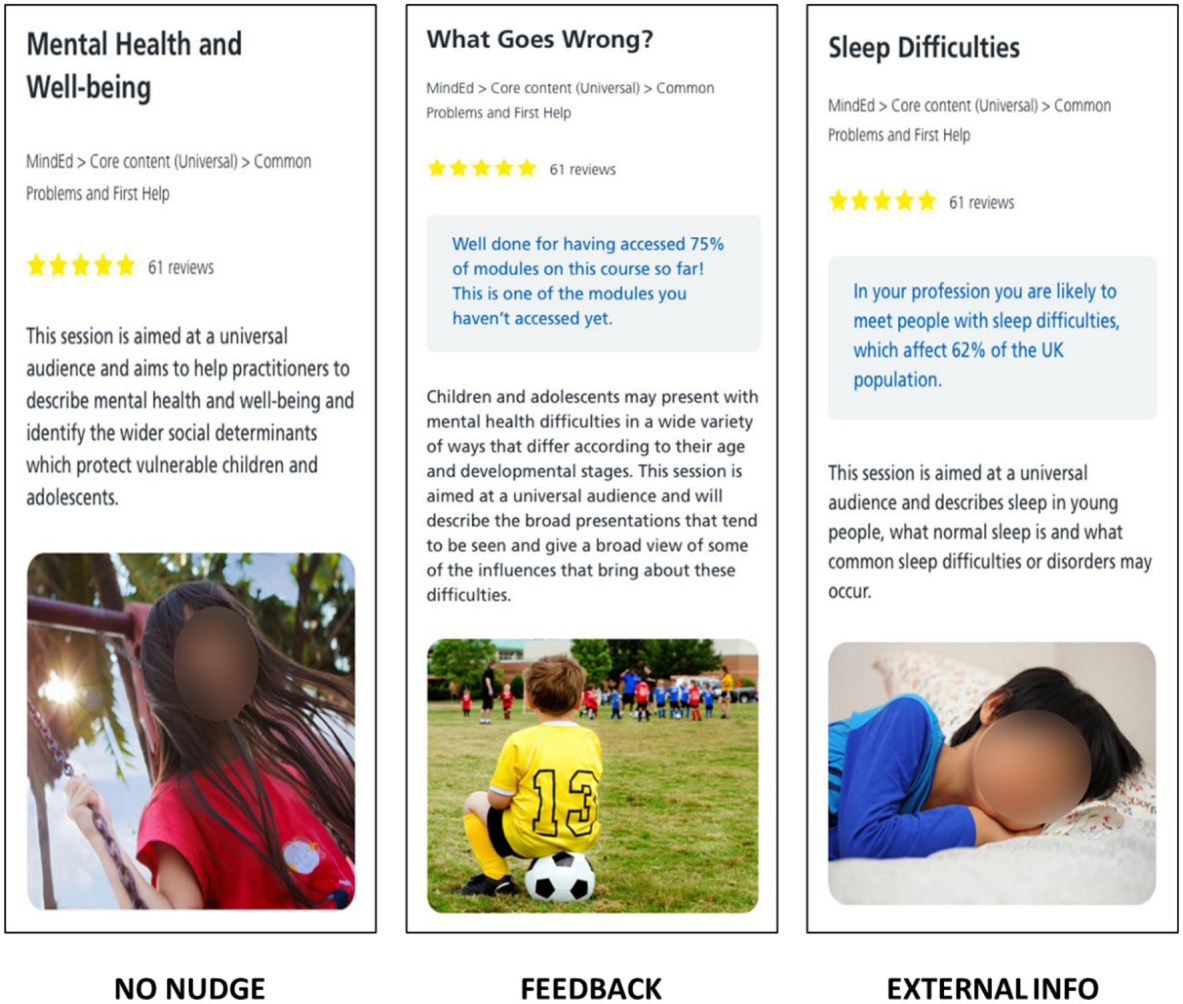
Digital nudges used in this research and presented to the participants in both user studies (No Nudge, Feedback, and External Information). The images show resources from e-Learning for Healthcare (https://www.e-lfh.org.uk/) with the corresponding nudges added to the interface (see blue text in the gray rectangles).

**Figure 4:**
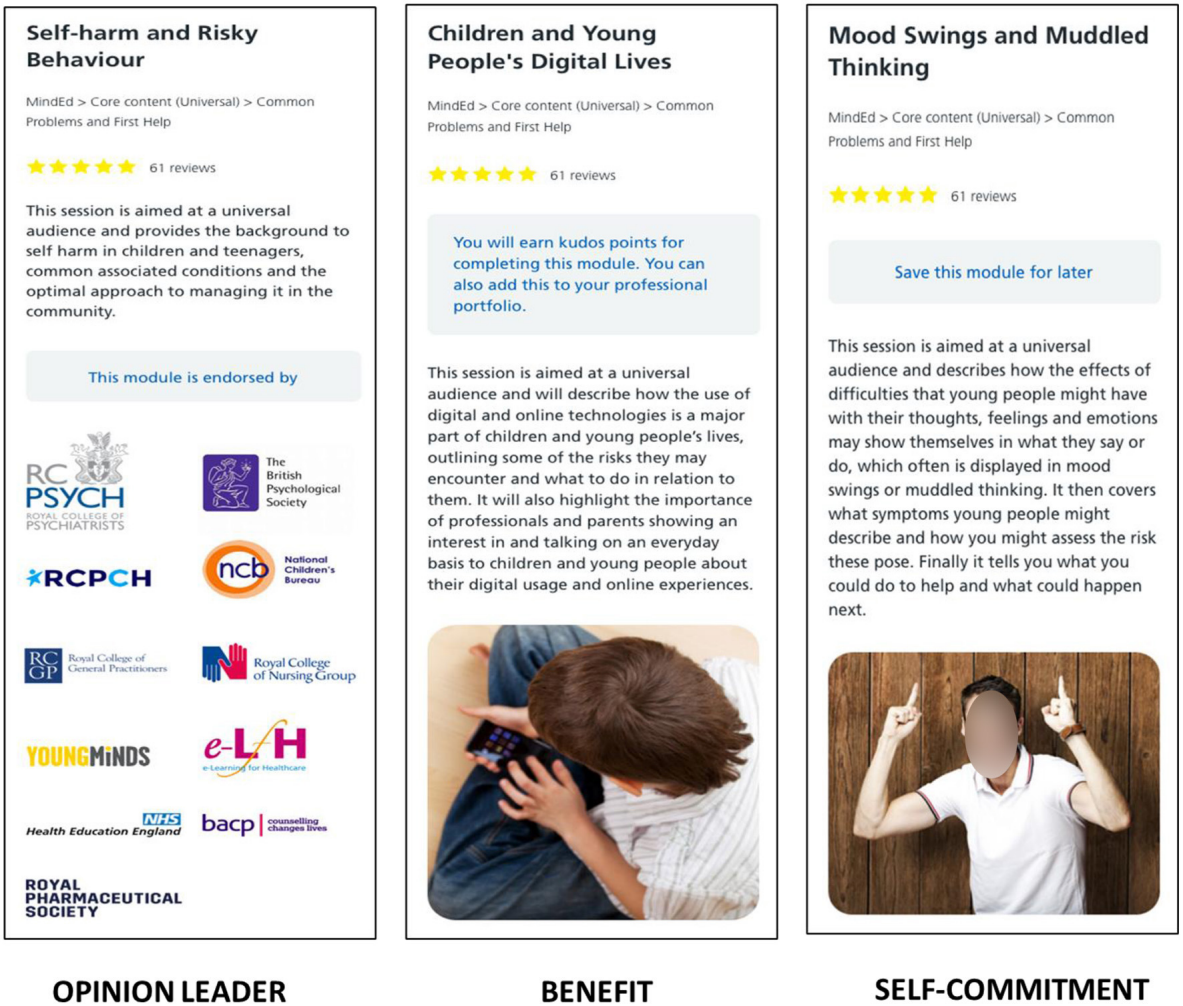
Digital nudges used in this research and presented to the participants in both user studies (opinion leader, benefit, and self-commitment). The images show resources from e-Learning for Healthcare (https://www.e-lfh.org.uk/) with the corresponding nudges added to the interface (see blue text in the gray rectangles).

**Part 4**. Selecting learning resources. The participants were presented with learning resources with nudges, as shown in Figures 3, 4. The following nudges were included:

- No-Nudge (the default interface offered in the HEE e-learning environment);
- Feedback (offers feedback on the learning progress);
- External information (offers additional facts that support the importance of the resource);
- Opinion Leader (provides the organizations that have endorsed the resource);
- Benefit (links to professional development and offers earning points, similar to “badges”);
- Self-commitment (provides an option to look at the resource later).

**Figure 5:**
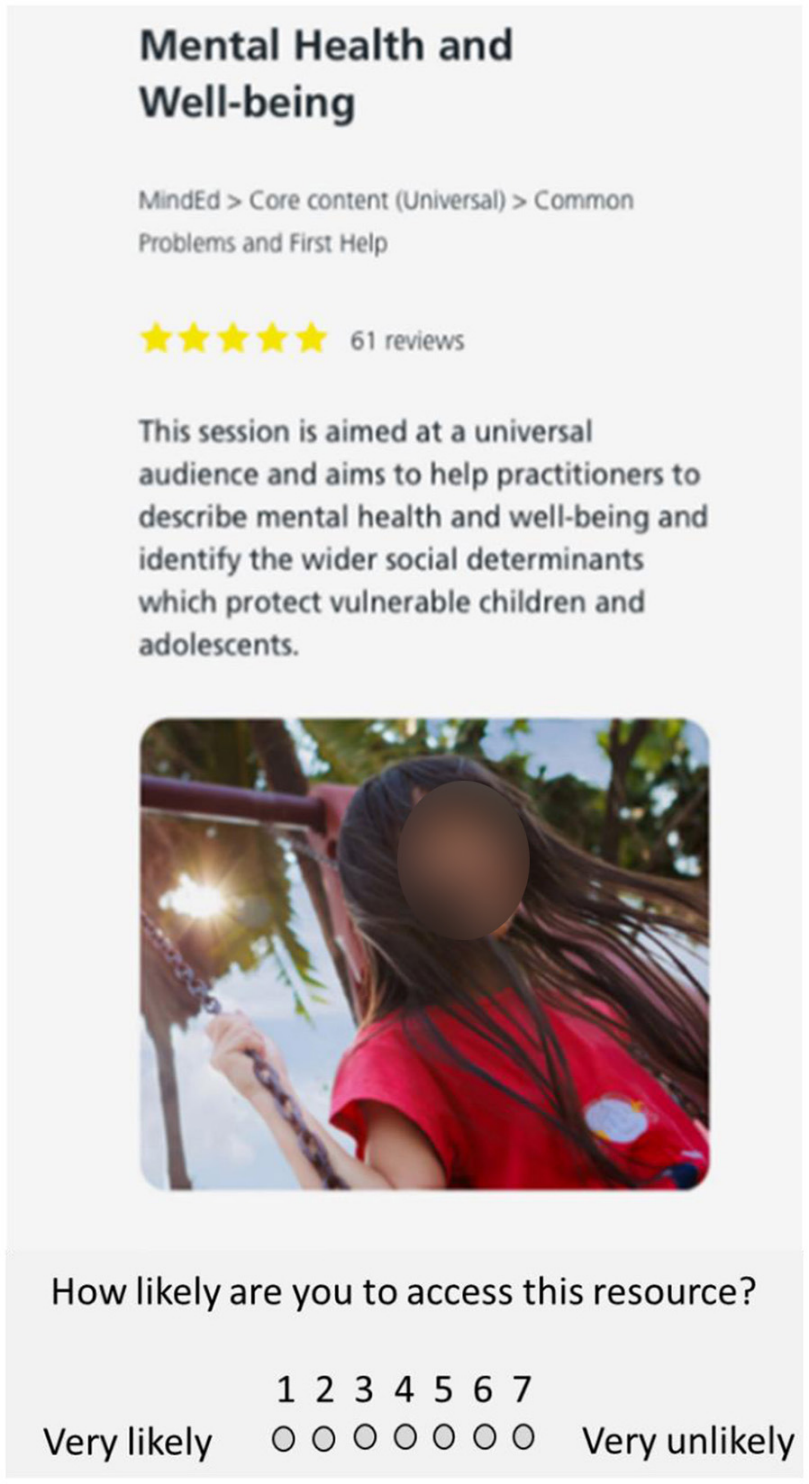
Example of the interface used for selecting a learning resource (in this example, NO NUDGE) asking the participants about the likelihood to select the resource. The image shows a resource from e-Learning for Healthcare (https://www.e-lfh.org.uk/) with the corresponding user feedback added at the bottom.

When presented with a learning resource, the participants were asked to indicate whether they are likely to access this resource (responding to a seven-point Likert scale; see Figure 5). An additional question encouraged them to briefly explain their decision to access the resource (the answers were not compulsory, but most participants provided some justification).

#### 3.3.3 Participants

In total, 157 users responded, including 123 from the first study (medical students) and 34 from the second study (healthcare professionals—people working in a large hospital in Leeds, UK). The first study (students) was mainly composed of individuals aged 17–34 years (23% healthcare professionals, 97% medical students), and the second study (healthcare professionals) included people aged older than 35 years (77% healthcare professionals, 3% medical students). In both cases, the use of technology for learning played a dominant role; however, a higher percentage of students use digital devices compared with healthcare professionals (86% and 52%, respectively; see Table 5).

**Table 5:** Use of technology among healthcare professionals and medical students.

**Description**	**Healthcare professionals (%)**	**Students (%)**
Daily	52	86
Several times a week	26	14
Several times a month	16	0
Several times a year	6	0

## 4 Results

### 4.1 Data integration

The first step of the analysis was to understand whether these two samples can be investigated as a single sample, despite the difference in characteristics such as age and occupation. To investigate such an aspect, a *t*-test analysis was conducted with Bonferroni–Holm correction to explore the scoring tendency in the likeliness to click for each resource (nudge). The results of *p*-values suggest that healthcare professionals and medical students did not differ significantly. Only in the case of the nudge labeled as “Opinion Leader”, the association is statistically significant (at *p* < 0.05) between the two studied groups (Table 6).

**Table 6:** Significance testing between medical students and healthcare professionals.

**Description**	**Interest (*p*-value)**	**Test value**	**CI**	**Likeliness (*p*-value)**	**Test value**	**CI**
No-nudge	> 0.05	0.46	(−0.32,0.53)	> 0.05	1.63	(−1.31,0.15)
Feedback	> 0.05	0.15	(−0.49,0.41)	> 0.05	1.29	(−1.15,0.27)
External information	> 0.05	0.62	(−0.31,0.62)	> 0.05	1.27	(−1.28,0.18)
Opinion leader	> 0.05	0.89	(−0.72,0.31)	< 0.05	2.96	(−1.86,−0.30)
Benefit	> 0.05	0.84	(−0.26,0.65)	> 0.05	0.45	(−0.53,0.85)
Self-commitment	> 0.05	0.85	(−0.45,0.54)	> 0.05	0.81	(−1.0,0.46)

The “Opinion Leader” nudge appears to have a significant impact on the likelihood of healthcare professionals and medical students clicking on a particular resource. When an association is statistically significant, the observed difference in behavior is not likely due to chance alone. To understand why this difference is significant, various factors can be considered. The “Opinion Leader” nudge, which conveys authority or expertise, resonates differently with healthcare professionals who may have a greater tendency to trust and follow expert opinions. In contrast, medical students who are still learning may need to be more receptive to authority figures. We have retained the data about the “Opinion Leader” nudge, as this provides valuable insights into how various demographic groups respond to different nudges, making it worth considering.

### 4.2 Personality insights extraction

The answers to open-ended questions stimulated participants to freely articulate their thoughts and feelings in their own words without the constraints of predefined response options. This provided text samples (min 500 words per participant) used as a source for extracting personality insights. From this, a word count process scrutinized 157 responses, ultimately identifying 140 as relevant due to the participants' willingness to provide detailed answers, with each response containing more than 100 words. The open-ended survey responses from each participant were analyzed using the Scaled Insights' Behavioral AI tool. This advanced tool can extract and analyze a wide range of personality traits, revealing 113 unique insights for each participant. Figure 2 visually displays the depth of information that was obtained from these open-text responses, indicating the abundance of insights that were gained. Furthermore, a clustering approach was employed to categorize participants into groups based on shared traits and behavioral patterns. This analytical technique provided a comprehensive understanding of the diverse personalities among the participant population.

### 4.3 Deriving user clusters

**Figure 6:**
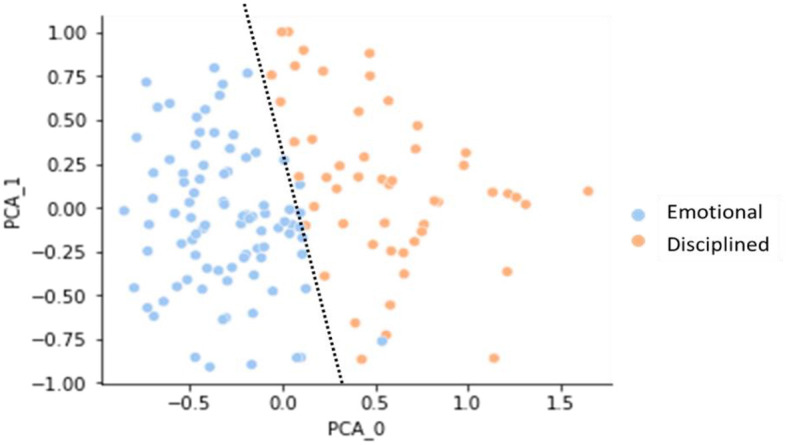
Personality clustering of medical students and healthcare professionals: insights from PCA and K-means algorithm.

In our analysis, we leveraged the power of the K-Means clustering algorithm to uncover underlying patterns in the personality scores of healthcare professionals and medical students, as shown in Table 6. The implementation was done in Python. The K-Means clustering algorithm allows us to automatically group participants into distinct clusters based on their personality traits. The K-Means algorithm begins by initializing k cluster centroids randomly, with k representing the number of desired clusters. It then iteratively refines these centroids by assigning data points to the cluster whose centroid is closest to them, calculating new centroids based on the mean of data points in each cluster and repeating this process until convergence. The resulting clusters, “Emotional” and “Disciplined,” are shown in Figure 6, providing valuable insights into each group's distinct personality traits, as presented in Table 7.

**Table 7:** Cluster centroids for the 10 insights with the most significant percentage difference.

**Personality Insights**	**“Emotional” cluster (*****N*** = **89)**		**“Disciplined” cluster (*****N*** = **51)**		**Percentage difference between Clusters**
	**Mean**	**Std dev**	**Mean**	**Std dev**	
Emotionality	0.58	0.14	0.32	0.14	58%
Modesty	0.39	0.15	0.23	0.12	53%
Social group orientated	0.43	0.16	0.28	0.15	43%
Distractible	0.53	0.09	0.35	0.08	40%
Self-discipline	0.34	0.10	0.50	0.14	36%
Openness	0.44	0.08	0.33	0.14	28%
Anxiety	0.57	0.09	0.43	0.14	27%
Vulnerability	0.62	0.09	0.47	0.14	27%
Workhorse	0.70	0.14	0.85	0.14	19%
Grounded	0.57	0.11	0.69	0.11	18%

The numerical values presented for personality insights correspond to the average score computed per insight using the Scaled Insights' Behavioral AI tool. The percentage difference (calculated as the difference between means divided by the average) indicates the differences between both clusters. The higher the percentage, the bigger the difference (i.e., the corresponding personality insights is more distinctive for the clusters).

**Figure 7:**
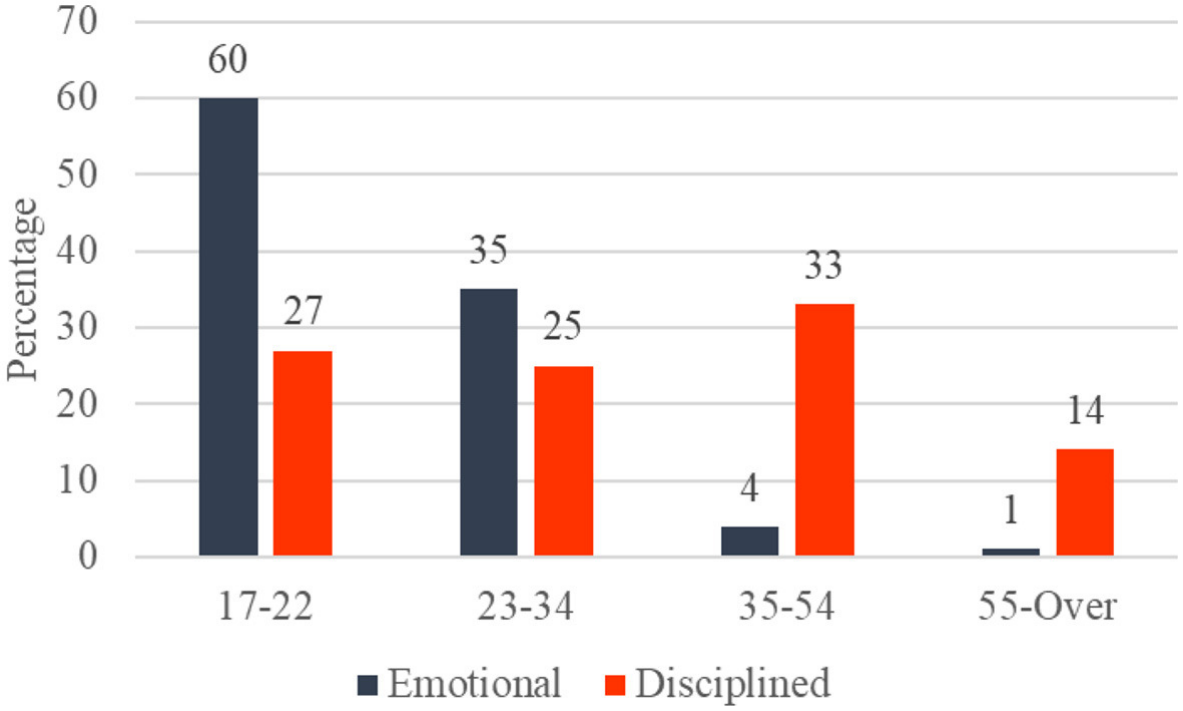
Distribution by age in the emotional and disciplined clusters.

We can also examine the personality clusters by their demographic information. Figure 7 shows age distribution; the “Emotional” group is mainly composed of individuals aged 17–22 years (60%). By contrast, the “Disciplined' one has a heterogeneous composition, mostly comprised of ages between 23 and 55 years (72%).

An intriguing pattern emerged that merits further investigation in analyzing the age distribution within the identified personality clusters. The “Emotional” group predominantly comprises individuals aged 17–22 years, constituting 60% of this cluster. Conversely, the “Disciplined” group displays a more diverse age composition, with the majority falling within the 23–55 age range, encompassing 72% of this cluster. While this observation implies a potential relation between age and personality traits, it is imperative to exercise caution when drawing definitive conclusions. Age represents just one facet of an individual's demographic profile, and personality traits are influenced by a complex interplay of factors, including life experiences, cultural background, and inherent individual differences. Therefore, although there seems to be a propensity for younger individuals to exhibit higher emotional traits and older individuals to gravitate toward discipline, it is essential to consider the multifaceted nature of personality development. Subsequent research endeavors could delve deeper into the intricate relationships between age, personality traits, and additional contextual variables, thereby contributing to a more comprehensive understanding of these dynamics.

### 4.4 Personality insights and nudges

This section examines how personality insights contribute to the nudge selection process by evaluating three additional user characteristics: interest, age, and use of Technology. The selection of such variables is because they can provide information on patterns linked to demographic data.

Tables 8, 9 show the results of the analysis. In the case of interest, age, and use of Technology, a linear regression model was used to predict the likeliness of clicking on each resource (No-Nudge, Feedback, External Information, Opinion Leader, Benefit, and Self-commitment) in both clusters: Disciplined and Emotional.

**Table 8:** R^2^ results for fitting linear regression.

**Description**	**Personality Insights**	**Interest**	**Age**	**Use of technology**
No-nudge	0.71	0.23	0.03	0.13
Feedback	0.59	0.24	0.19	0.08
External information	0.70	0.35	0.10	0.06
Opinion leader	0.73	0.33	0.02	0.08
Benefit	0.74	0.28	0.01	0.03
Self-commitment	0.70	0.39	0.01	0.14

The numerical values presented for personality insights indicate the likelihood of selecting a resource based on multiple variables, such as Personality Insights, Interests, Age, and Use of Technology within the 'Disciplined' cluster.

**Table 9:** Linear regression analysis of resource selection in emotional cluster based on multiple variables.

**Description**	**Personality insights**	**Interest**	**Age**	**Use of technology**
No-nudge	0.46	0.33	0.05	0.04
Feedback	0.31	0.43	0.05	0.03
External information	0.37	0.44	0.01	0.05
Opinion leader	0.54	0.33	0.05	0.06
Benefit	0.31	0.38	0.02	0.06
Self-commitment	0.34	0.40	0.02	0.09

Regarding personality insights, 113 personality insights are calculated for each cluster (Emotional and Disciplined). The percentage difference is computed between the clusters (shown in Table 10). A multiple linear regression model has been used to predict the likeliness of clicking on each resource.

**Table 10:** Cluster centroids for the ten insights with the most significant percentage difference in the emotional group.

**Personality insights**	**Emotional “A” (*****N*** = **49)**		**Emotional “B” (*****N*** = **40)**		**Percentage difference between Clusters**
	**Mean**	**Std Dev**	**Mean**	**Std Dev**	
Happiness	0.35	0.08	0.48	0.11	32%
Neuroticism	0.49	0.19	0.36	0.22	30%
Aggressive	0.36	0.10	0.26	0.10	30%
Cold	0.48	0.11	0.37	0.11	25%
Persuasive	0.49	0.11	0.63	0.09	24%
Self-Discipline	0.31	0.09	0.39	0.09	24%
Depression	0.37	0.10	0.29	0.08	22%
Social group orientated	0.39	0.16	0.48	0.15	21%
Body Focus	0.71	0.14	0.59	0.20	19%
Emotionality	0.53	0.15	0.65	0.10	19%

^*^The numerical values for personality insights correspond to the average computed score per insight using the Scaled Insights' Behavioral AI tool. The percentage difference (calculated as the difference between means divided by the average) indicates the differences between both clusters. The higher the percentage, the bigger the difference (i.e., the corresponding personality insight is more distinctive for the clusters).

**Figure 8:**
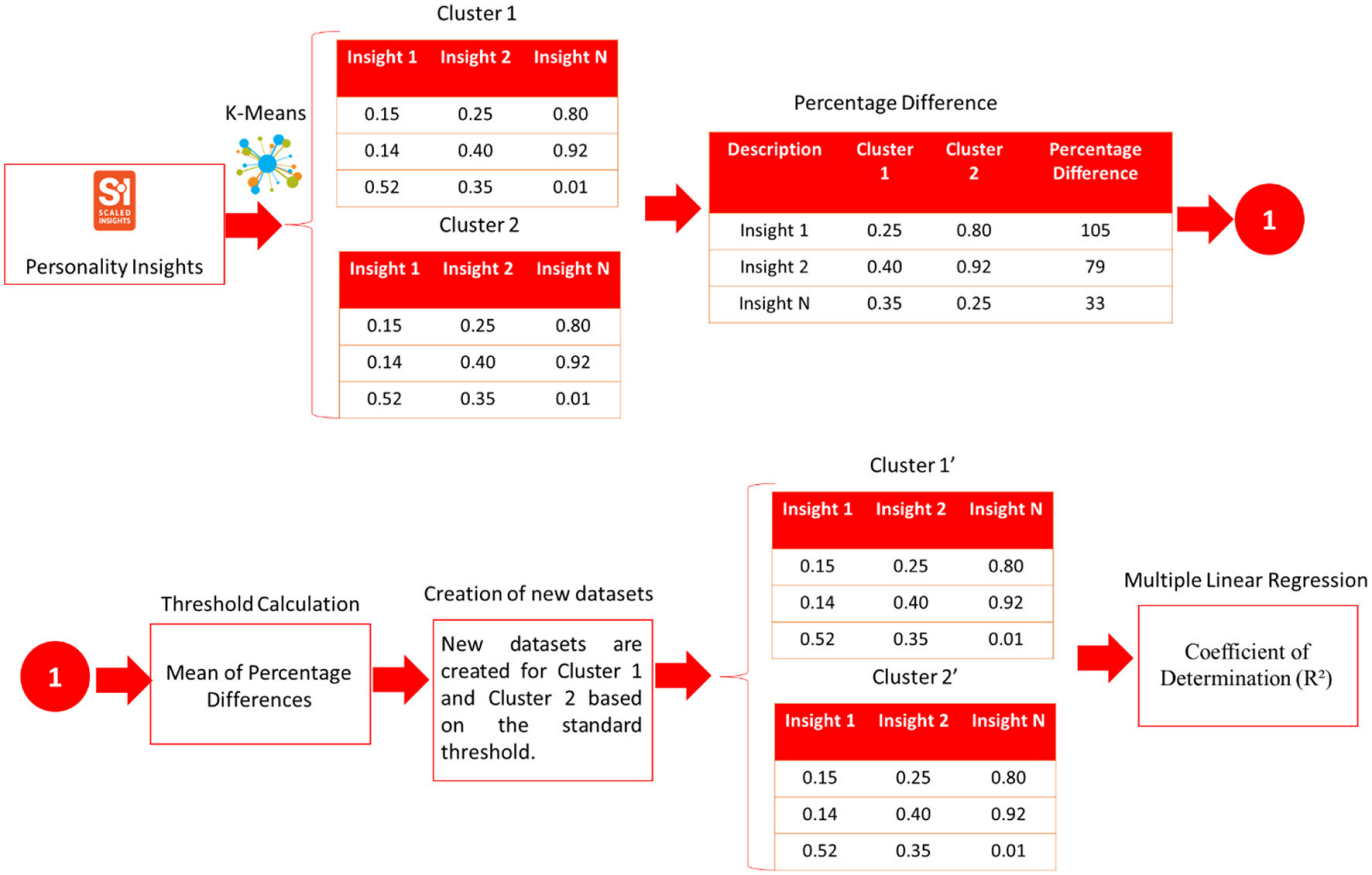
Workflow for the multiple linear regression process for personality insights.

Several steps are followed to select which personality insights can feed the multiple linear regression model based on their importance. First, the mean of the percentage differences of the two clusters is calculated to set a standard threshold; then, only those personality insights whose percentage difference is above the precomputed verge are selected. Second, new datasets are created for both clusters based on the preliminary step. Third, the new datasets are ingested into the regression model to calculate the coefficient of determination (R^2^) (see Figure 8, Tables 8, 9).

In the “Disciplined” group, the coefficient of determination (R^2^) results suggest that personality insights outperformed the other examined variables, such as interest, age, and use of Technology, and they represent a characteristic that could influence the likeliness of selecting a specific nudge. In addition, to measure the association between personality insights and interest, the Pearson correlation index was calculated. A result of 0.35 suggests that individuals were attracted to selecting the presented resources.

In the “Emotional” group, two main aspects need to be outlined. First, the coefficient of determination (R^2^) results displayed lower scores in comparison with the “Disciplined” group. Second, and in a similar fashion to the previous paragraph, the Pearson correlation index was computed between personality insights and interest. A negative outcome of −0.77 indicates a low engagement rate (disinterest) of individuals; hence, users are unlikely to select the presented resources.

Given the previously identified low engagement rate in the “Emotional” group, it is important to explore whether additional personality insights could have influenced this cluster. This could offer valuable insights into the effectiveness of nudges aimed at improving engagement levels.

**Figure 9:**
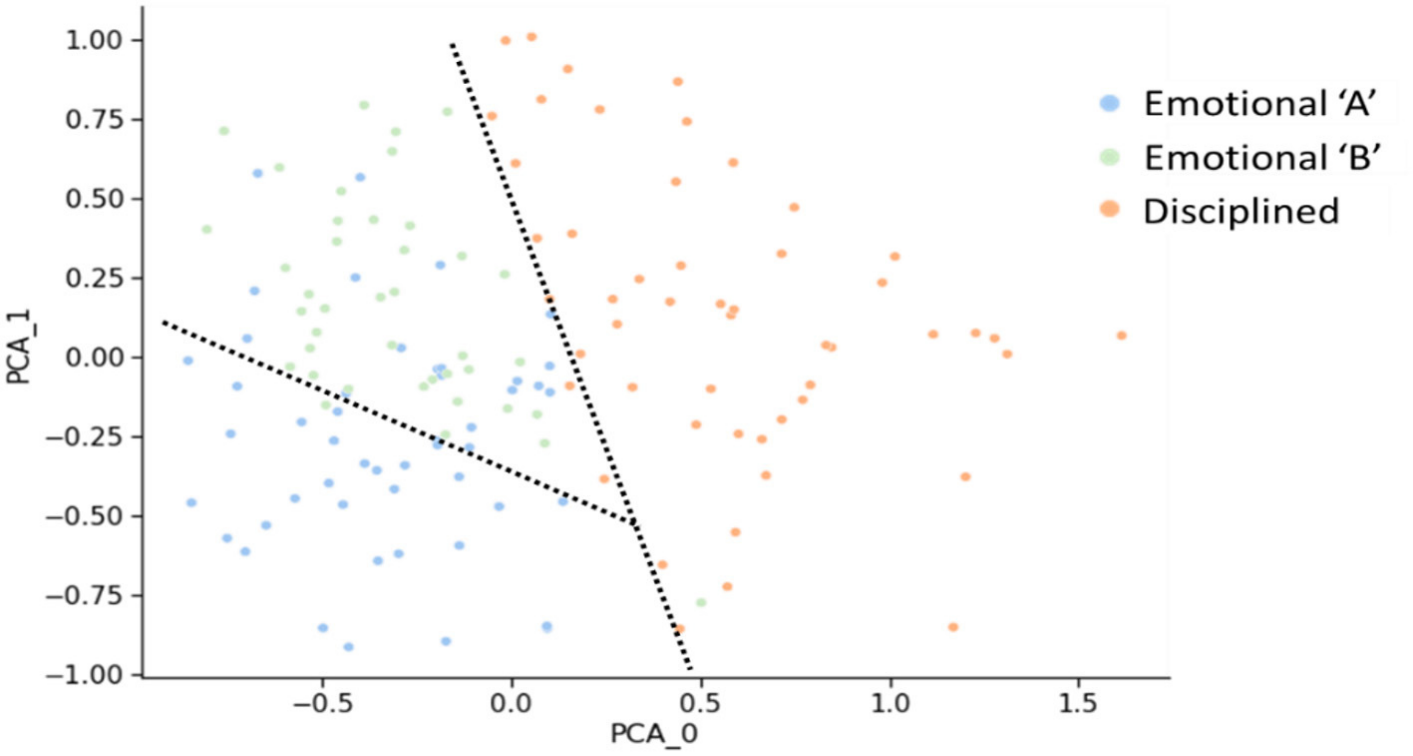
Personality clusters of medical students and healthcare professionals: a PCA visualization of emotional A, emotional B, and disciplined groups.

A centroid-based algorithm was used (K-Means) to spot new sub-clusters (Figure 9). Two new subgroups were identified, which were characterized by different personality insights (Table 10). To illustrate such personality differences, the group labeled as Emotional “A” was represented by relatively higher mean scores on neuroticism, aggressiveness, cold, depression, and body focus. By contrast, the set tagged as Emotional “B” presented relatively higher mean scores on happiness, persuasiveness, self-discipline, social group-oriented, and emotionality.

It should be noted that the personality insights that described the Emotional “B” group are related to optimism and persuasion, among others (Table 10). By contrast, the Emotional “A” group is overall characterized by aggressiveness and difficulty empathizing with others. Such personality differences suggest that individuals are less likely to select the nudges presented during this study.

As discussed previously, personality insights can provide valuable information to inform the selection of nudges for recommending educational resources. The findings suggest that personality insights outperformed other examined variables such as interest, age, and use of technology, particularly in the “Disciplined” group. Conversely, the “Emotional” group displayed a low level of engagement. The results demonstrate that personality insights can offer a deeper understanding of user behavior and preferences, which can help to enhance the effectiveness of nudges in recommending educational resources. Therefore, incorporating personality insights into the nudge selection process could be a promising approach to personalize educational recommendations and improve user engagement.

## 5 Discussion and conclusion

The study presented here investigated the role of personality insights when recommending resources for learning in the healthcare domain. Specifically, the study examined how individual differences, including personality insights, interest, age, and use of technology, predict the effectiveness of nudges for e-learning resources among healthcare professionals and medical students.

Two user studies with medical students and healthcare professionals were combined. The findings showed positive evidence that personality could work as a possible predictor when recommending resources for informal learning. Other variables, such as age, use of technology, and interest, did not appear to influence the nudge prediction process. The study used 113 personality insights, including the Big Five, Big Five Facets, Basic Human Values, and Needs, to create clusters based on personality and analyze how they impact the selection of resources.

The use of a cluster-based approach helped to identify the inherent resource preferences of different types of people based on their individual personality characteristics. This approach provides a more comprehensive understanding of how personality influences resource selection, as it considers the diversity of personality insights rather than just the Big Five. The use of the Scaled Insights' Behavioral AI tool provided a rich set of personality insights (113) and allowed the findings to be enriched the analysis compared with those studies that were limited to the Big Five model.

### 5.1 Implications for using nudges for recommending educational resources

The findings of the two user studies have several implications for using nudges for educational resources. First, the results suggest that personality insights could be a useful tool for personalizing the selection of resources for self-learning. While the findings here are for healthcare education, they indicate that in the broader context, considering personality insights can be helpful for designing nudges for learning and educational interventions, in general. Personalization of the resources based on an individual's personality could increase their engagement and motivation toward the learning process.

Second, the results suggest that individual differences are important for predicting the selection of resources and designing nudges when recommending resources. While the findings show that in the specific experimental context, age, use of technology, and interest are weaker than personality insights when predicting the use of nudges to use educational resources, this could differ in other contexts. However, the results of our study show that interventions and resources that are tailored to individual personalities would be effective (and in some cases even more effective than demographic characteristics). This points toward the need to consider a range of individual differences, such as personality.

Third, the user studies shed light on the people's motivations to select certain nudges. Analysis of qualitative data (when participants briefly explained why they would select or not select a resource) indicated that participants had different motivations when selecting nudges.

User interests (related to their immediate job or their aspirations to improve) influenced the selection of nudges. The following user comments illustrate this:

- Reasons to select the resource: “*(the resources topic)…is a subject that might prove helpful in terms of my voluntary role”[Study2-P3]*; “*It is useful both personally and professionally to have an understanding of the topic”[Study1-P85]*; “*Relevant to me as a parent of teenagers”[Study2-P12]*; “*I would click on this resource as it is an important area of mental health”[Study1-P94]*; “*It sounds very relevant to modern life and is an important topic to have awareness about”[Study2-P18]*.
- Reasons not to select the resource: “*Not interested in this topic and outside field of work”[Study2-P14]*; “*Not interested in sleep medicine”[Study2-P29]*.

Therefore, although personality can influence the choices of nudges, it should not be observed as the sole parameter used for personalization. The user's interest in the topic should not be ignored but should be considered together with personality. If a user is interested in a topic, there may be less need for persuasion; while if the user does not have explicit interest in the topic, subtle nudges to accompany the recommendation (e.g., indicating others' ratings or providing cues about the value of the resource) would be beneficial.

Another factor that plays a role when selecting nudges is the quality of the resource and the use of visual effects (e.g., catchy title, attracting images). The following user comments illustrate this:

- Reasons to select the resource: “*The photo intrigues me, and does make me want to know more.”[Study2-P17]*; “*The image speaks directly to the topic”[Study2-P34]*.
- Reasons not to select the resource: “*This resource could be useful if it describes more uncommon presentations.”[Study1-P3]; “…the introduction is just a big block of text with long words. It's hard going to read it.”[Study2-P17]; “The explanation was too wordy didn't draw you in.”[Study2-P30]*.

This indicates that the effect of personalization features (like nudges in this case) will always depend on the quality of the resources. Crucially, the resource title and the additional media used could play the role of nudges, if properly designed, or could deter the users from the resources. We tried to select HEE resources that were similar, all on important topics for healthcare professionals, and all linked to professional development. Despite this, the participants noted differences in the effect of titles and images. Further research is needed to explore how to use resource titles, summary, and images to nudge the user to select the resource. Studies with news articles (Piotrkowicz et al., 2020) indicate that sentiment and linguistic style of news titles will have an impact on selecting news articles.

Crucially, some responses to clarify the selection can be linked to the information shown in the nudge. This is important, as the nudges were carefully designed with experts in psychology and behavior change (Piotrkowicz et al., 2020) to offer subtle cues without directly suggesting that the resource should be read. The following user comments illustrate that the users have noted the nudges:

- Feedback nudge: Several users noted the nudge, *e.g. “Having a prompt showing that this is a resource required for me to finish my training is beneficial and would make me click on it”[Study2-P15]; “You can be overwhelmed by e-learning modules not knowing how much you have completed is good.”[Study2-P18]*.
- External information nudge: Only one user noted the *nudge ”The additional fact in the information makes it more relatable and increase my interest in learning further.”[Study1-P18]*, which indicate the low interest in using this nudge.
- Opinion leader nudge: Two-thirds of the medical students pointed that the endorsement of the resource by the relevant professional bodies was the reason to be willing to select the recourse: “*Well endorsed by highly regarded regulators”[Study1-P4]; “The endorsements from professional bodies helps significantly”[Study1-P1]. “Knowing that the content of the module has been validated by professionals is a major selling point” [Study1-P2]*. In contrast, only 6 out of 34 (17%) of healthcare professionals noted the endorsement, 3 noted in a positive way, e.g., “*By having the endorsements- it provides reassurance about quality of the session*”*[Study2-P19]* and 3 noted in a negative way, e.g., “*All the logos clutter the page and don't really mean much”[Study2-P15]*. Hence, the Opinion leader nudge appears more effective when the learners are less experienced in the domain. In these cases, having validation of the resource from someone they respect would be helpful.
- Benefit nudge: All together five users noted the nudge when justifying their decision to select/or not select the resource, and all these were negative about the nudge, e.g., “*The word kudos put me off if I am honest.” [Study1-P11]; “Not bothered about kudos.” [Study2-P19]*. This suggests that the formulation of benefits may have put people off. Further exploration is needed to identify how to shape the Benefits nudge, e.g., building on research on badges and rewards.
- Self-commitment nudge: Only three healthcare professionals noted the nudge, e.g., “*Saving for later is quick and easy and would make me more likely to come back to it if I didn't have the opportunity to complete it at the time”[Study2-P14]*. This points at self-regulation and planning abilities, which healthcare professionals have developed but medical students may be lacking. In the current design, the nudge would be beneficial for people with self-regulation abilities. The nudge can be used as a vehicle to develop self-regulation but would need to be combined with additional description.

### 5.2 Conceptual framework for personalizing nudges based on personality traits

In the context of digital health interventions and e-learning, the present study highlights the pivotal role of personalization, particularly concerning the influence of personality traits on the effectiveness of nudges. To provide practical insights for researchers, designers, and practitioners, we propose a pipeline for personalizing nudges based on personality insights. This pipeline aims to bridge the gap between theory and application, ensuring the efficient integration of personality insights into designing effective digital interventions for learning. It includes the following steps:

- **Step 1: Understanding the Multifaceted Nature of Personality:** To create effective personalization, it is essential to go beyond the traditional Big Five personality traits and consider the multifaceted nature of personality. Researchers and designers could explore a wide range of personality insights, such as the Big Five Facets, Basic Human Values, and Needs, among others, to create a comprehensive personality profile. This foundational understanding could be crucial for successful personalization.
- **Step 2: Dynamic Personality Clusters:** Building on this foundation, the proposed framework advocates for creating dynamic personality clusters using a well-defined process. This process comprises the following sub-steps:
  - **Sample Collection:** This step aims to collect a language sample and specific behaviors from the target user group. This can be done through a variety of methods, such as surveys or interviews. The goal is to collect a large enough sample that is representative of the target population.
  - **Detect personality:** The second step focuses on utilizing AI-driven analysis tools to discover common personality traits and specific behavioral attributes within the sample. These tools can be used to analyze the language sample and specific behaviors in order to identify patterns and correlations. We have used the Scaled Insights behavior tool to analyze textual samples. Other approaches can also be used (e.g., eye tracking).
- **Step 3: Mapping**: The third step centers on grouping individuals with common personality characteristics and behaviors into distinct personality clusters. It also includes the development of ground truth dataset that informs how a new individual with specific personality traits will likely behave in a given context.
- **Step 4: Nudging**: The following phase is collaborating with experienced professionals and behavior change experts to design nudges tailored to each identified personality cluster. These nudges should be optimized for shifting behavior change effectively.

### 5.3 Comparison with previous studies

Previous research in the field of personalized e-learning and recommendation systems has highlighted the importance of considering individual differences, including personality insights, in the design of educational interventions and resources. Studies such as those conducted by Kew and Tasir (2022) have shown that personalizing e-learning systems based on individual characteristics can increase engagement and motivation toward the learning process. However, the scope of this study goes further by investigating the specific role of personality insights in recommending resources for learning in the healthcare domain.

In a meta-analysis on the effects of personality on academic outcomes (Poropat, 2009), various studies were reviewed that examined the relationship between personality insights and academic performance. Poropat (2009) found that certain personality insights, such as conscientiousness and openness to experience, were positively associated with academic achievement. More recently, studies (Souabi et al., 2021; Zhang et al., 2021; Lu and Kan, 2023) have explored the role of personality insights in the design of e-learning systems and recommendation systems, finding that incorporating personality insights can lead to more effective and personalized interventions. The study presented here contributes to this area of research. The present study utilized a cluster-based approach and a Behavioral AI tool developed by Scaled Insights to investigate the influence of a broader range of personality insights on resource selection in e-learning systems.

This study has advanced efforts to use behavioral nudges to engage and increase the uptake of educational training. This study offers insights into the use of a unique behavioral AI method that infers personality attributes and features from natural language construction. Hence, it can provide a rapid method for digital education providers to nudge learners more effectively by aligning nudges with individuals' personality attributes. Further research is needed that extends and teases out the effects observed in this study.

### 5.4 Limitations and future research

Before closing the discussion section, it is essential to describe the limitations and future research areas of the present study. First, the study relied on self-reported data, where participants were asked about which resources they would select rather than examining their actual resource selection history. While this is a common method to investigate user acceptance of nudges, it is observed as a starting step to indicate the importance of personality insights. Further research will include adding nudges to the recommender system and exploring the effect of personality on the selection of resources. The methodology in the study by Dimitrova and Mitrovic (2021) can be a useful starting point for further investigation.

Second, the sample size could have a significant impact, particularly on the group of healthcare professionals. This was due to the timing of the study coinciding with the COVID-19 pandemic, which made it difficult for such a group of people to participate because of their heavy workloads. In addition, research linked to work-based learning and involving professionals is always challenging, especially when these people are busy at work and do not have specific incentives to take part in research studies. Despite the limited size, the findings still provide valuable insights into the resource preferences and personalities of experienced professionals in the healthcare context.

Third, while using personality clusters allows integration of a breadth of personality insights, it also can lead to some group bias. For example, there is no clear separation in the clustering results (especially the second Emotional cluster in Figure 9). When conclusions are drawn at a cluster level, the results may be biased toward most individuals falling in the cluster and may not account for those users who are misclassified. This is a common issue with group-level (stereotype-based) personalization. To reduce possible bias, we would suggest that (a) personality clusters are combined with other user characteristics, e.g., interests and experience; and (b) a broader range of nudges are offered so that the users are not disadvantaged.

Fourth, the study focuses on the healthcare domain and the informational content of the HEE learning platform. Therefore, the findings may not be generalizable to other domains or learning contexts. Additionally, the analysis was conducted using Scaled Insights' Behavior AI tool trained in English, which may limit the generalizability of the results to other languages. Further research is needed to examine the impact of personality insights in other educational contexts and with other languages. Further research is needed that investigates the role of personality insights in other contexts or to include other factors that could influence the acceptance of nudges. Finally, the study was conducted in a specific language (English) and culture (British), and the results may not be generalizable to other languages and cultures.

Future research is needed to investigate the impact of different types of multimedia, such as videos and images, on the nudge selection process using personality insights as the primary predictor. The inclusion of videos could be explored in terms of length and participant comments to gain insight into the preferences of different personality clusters. Another area for future research is to study the behavior of “gray sheep” users, who are known to have unique tastes and preferences and are challenging to nudge. The use of personality insights could be analyzed to determine the extent to which they could contribute to understanding these specific choices.

### 5.5 Conclusion

This research provides valuable insights into how individual differences, including personality insights, impact the selection of resources for self-learning in the healthcare domain. The findings suggest that personality insights could be a useful tool for personalizing nudges for selecting resources. The study also points toward the need for more research to understand the impact of individual differences, including personality insights, on designing nudges for learning.

In this study, the use of a cluster-based approach and the Behavior AI tool developed by Scaled Insights provides a more comprehensive understanding of how personality influences resource selection by considering the diversity of personality insights. The results of the study have the potential to inform the design of more effective and engaging e-learning platforms for medical students and healthcare professionals, ultimately contributing to the advancement of healthcare education. In a broader context, our study shows that personality insights could be considered when designing interventions in e-learning.

## Data availability statement

The original contributions presented in the study are included in the article/supplementary material, further inquiries can be directed to the corresponding author.

## Ethics statement

Ethical approval was obtained from the Faculty of Medicine and Health, University of Leeds, UK for both studies. For Study 2, which involved health professionals, ethical approval was obtained also from the UK NHS Research Ethics Committee.

## Author contributions

SF, VD, SS, and PC contributed to the conception and design of the study. All authors contributed to manuscript revision, read, and approved the submitted version.

## Footnotes

^1^Statista. (n.d.). E-learning: taking courses online in Great Britain. Retrieved from https://www.statista.com/statistics/286318/e-learning-taking-courses-online-in-great-britain/ (Statistica, 2022a,d).

^2^Statista. (n.d.). FutureLearn course registrations. Retrieved from https://www.statista.com/statistics/882141/futurelearn-course-registrations/ (Statistica, 2022b,c; Tkalcic and Vozab, 2020).

^3^Health Education England. (n.d.). Health Education England. Retrieved from https://www.hee.nhs.uk/ (HEE, 2022b).

^4^e-LfH. (n.d.). e-Learning for Healthcare. Retrieved from https://www.e-lfh.org.uk/ (HEE, 2022a).

^5^In this study, we use the term “personality insights”, instead of the more popularly used terms “personality traits” or “personality types”, to note the broader nature of the insights captured, including both Big Five and Human Values.

^6^Scaled Insights. (n.d.). Retrieved from https://www.scaledinsights.com (Scaled Insights, 2022).
